# What's in a Typeface? Evidence of the Existence of Print Personalities in Arabic

**DOI:** 10.3389/fpsyg.2017.01229

**Published:** 2017-10-18

**Authors:** Timothy R. Jordan, Alya S. AlShamsi, Hajar A. K. Yekani, Maryam AlJassmi, Nada Al Dosari, Ehab W. Hermena, Mercedes Sheen

**Affiliations:** Department of Psychology, Zayed University, Dubai, United Arab Emirates

**Keywords:** reading, printing, personality, vision, text

## Abstract

Previous research suggests that different typefaces can be perceived as having distinct personality characteristics (such as strength, elegance, friendliness, romance, and humor) and that these “print personalities” elicit information in the reader that is in addition to the meaning conveyed linguistically by words. However, research in this area has previously been conducted using only English stimuli and so it may be that typefaces in English, and other languages using the Latinate alphabet, lend themselves unusually well to eliciting perception of print personalities, and the phenomenon is not a language universal. But not all written languages are Latinate languages, and one language that is especially visually distinct is Arabic. In particular, apart from being read from right to left, Arabic is formed in a cursive script in which the visual appearance of letters contrasts strongly with those used for Latinate languages. In addition, spaces between letters seldom exist in Arabic and the visual appearance of even the same letters can vary considerably within the same typeface depending on their contextual location within a word. Accordingly, the purpose of the present study was to investigate whether, like English, different Arabic typefaces inspire the attribution of print personalities. Eleven different typefaces were presented in Arabic sentences to skilled readers of Arabic and participants rated each typeface according to 20 different personality characteristics. The results showed that each typeface produced a different pattern of ratings of personality characteristics and suggest that, like English, Arabic typefaces are perceived as having distinct print personalities. Some of the implications of these results for the processes involved in reading are discussed.

## Introduction

Previous research has revealed that different typefaces are often perceived as having visible personality traits (which we call *print personalities*) with the ability to convey semantic information beyond the meaning provided linguistically by the words themselves. Over time, this capacity for typefaces to elicit feelings in readers has been referred to variously as atmosphere value (Poffenberger and Franken, 1923), congeniality (Zachrisson, 1965), semantic quality (Bartram, 1982), topographical allusion (Lewis and Walker, 1989), personality (Striver, 2001), and rhetorical effects (Mackiewicz and Moeller, 2004). Indeed, several reports have argued that the visual attributes of written words have a subtle influence on perception extending beyond matters of legibility (e.g., Kostelnick, 1990; Brumberger, 2003; Mackiewicz, 2004). Sushan and Wright (1989), for example, claim that each typeface has a discrete personality and can be characterized in many ways, including confident, elegant, casual, bold, romantic, friendly, nostalgic, modern, delicate, and sassy, with as many potential personalities as there are actual typefaces. In addition, Parker (1997) proposed that typefaces with rounded serifs are perceived as friendly and open whilst typefaces with square serifs are perceived as formal and proper. Whether or not a typeface has a serif also affects the number of affective characteristics ascribed to it, with serif typefaces eliciting more emotion-laden adjectives than sans serif typefaces (Tantillo et al., 1995). Serif typefaces, for example, include 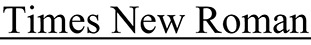, which is typically described as reliable and bookish, 
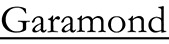
, which is described as graceful, refined, and feminine, and 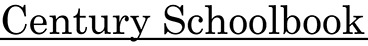, which is described as serious but friendly, whereas 
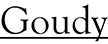
 is podgy, jolly and without pretension (Secrest, 1947), and the 
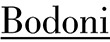
 family of type is dramatic, sophisticated and urbane (Sushan and Wright, 1989). On the other hand, sans serif fonts elicit fewer attributes generally, but they are nonetheless still perceived as having distinct personalities. 
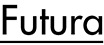
, for example, is described as no-nonsense and restrained, whereas 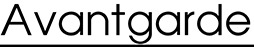 is modern and cool (Spiekermann and Ginger, 1993). In a similar vein, typefaces that are lighter in weight (i.e., have thinner strokes) are perceived as delicate, gentle, and feminine, whilst heavier fonts (with thicker strokes) are perceived as strong, aggressive, and masculine, and even adding longer ascenders and descenders to different typefaces can transform the print personality perceived (Sassoon, 1993).

Research investigating the interaction between print personality and word meaning has shown that when typeface and word meaning are congruent (e.g., the word “reliable” is presented in 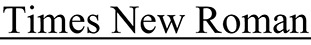) as opposed to incongruent (e.g., the word “romantic” presented in 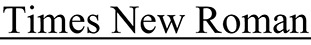), words are read with greater fluency, word meanings are reported more efficiently, and the message is considered to be more sincere (e.g., Lewis and Walker, 1989; Doyle and Bottomley, 2004; Oppenheimer and Frank, 2008). Indeed, using the right typeface for the right purpose appears to have important implications not only for legibility (Zachrisson, 1965) but also for factors such as comprehension (Lewis and Walker, 1989), memorability (Poffenberger and Franken, 1923), priming (Fazio, 2001), persuasion (McCarthy and Mothersbaugh, 2002; Juni and Gross, 2008), perceptual fluency (Oppenheimer and Frank, 2008), and even motivation to exercise (Song and Schwarz, 2008).

From this body of work, it seems that the surface details of typefaces are encoded in a way that conveys meaning generated directly by the typeface's visual structure. At the simplest level, there may be a direct correspondence between a typeface's physical characteristics and its perceived personality; for example, a typeface in bold may inspire perceived qualities such as thickness and volume, or even heaviness and density. But less easily explicable are the links between typefaces and other more abstract connotative dimensions, such as confident, casual, or romantic. Presumably, judgments in these cases are mediated by the perceived similarity of a typeface's visual form to objects in the real world which possess such qualities (see Lewis and Walker, 1989).

However, while it has been known for many years that typefaces are often assigned personalities (e.g., Poffenberger and Franken, 1923), this work has so far been reported for stimuli printed only in English, using the Latinate alphabet. Accordingly, it remains to be determined whether the formation of print personalities is confined to languages possessing only certain visual characteristics, or is a more general component of language perception. Of particular relevance is that letters in languages using the Latinate alphabet are usually physically distinct and physically consistent within a particular typeface. The letters on this page, for example, are each separated by clear spacing and are physically identical irrespective of where in a word they occur (with the occasional exception of initial letters that are capitalized). This aspect of printed text makes the physical characteristics of each letter in a typeface readily apparent. As a result, if the formation of print personalities relies on clearly perceiving the consistent physical form of letters, Latinate text may be unusually well-suited to producing this phenomenon. But because research into print personality so far has used only the Latinate alphabet, it is currently not known whether letter discriminability and consistency in languages is necessary for the formation of the phenomenon.

Although research into print personality has not addressed this issue, findings from the word recognition literature do suggest that variations in the discriminability and physical form of letters can impair how words are perceived. For example, several studies have shown that alternating the case in which letters are pRiNtEd disrupts word recognition observed for normal, consistent uppercase and lowercase stimuli (e.g., Mayall et al., 1997; Jordan et al., 2003; see also Juhasz et al., 2006) and there is good evidence to suggest that these effects of varying the appearance of letters are produced by disrupting the visual processing of stimuli (e.g., Perea et al., 2015; see also Mayall et al., 1997). Indeed, word recognition seems to be sensitive to the nature of the typeface in which words are presented, and even the presence of serifs can affect the identification of word stimuli (e.g., Moret-Tatay and Perea, 2011). The relationship between these effects of the discriminability and physical form of letters on word perception and the formation of print personalities is currently unknown. But if visual discriminability and consistency are important for producing print personalities, typefaces in English, and in other Latinate languages, may lend themselves unusually well to eliciting these abstract sources of meaning. Accordingly, further evidence is required to indicate whether or not print personalities may be a universal characteristic of written language perception.

Not all written languages are like Latinate languages, and one language that is particularly visually distinct is Arabic. Arabic has the second-most used alphabet in human societies, after the Latinate alphabet. But unlike languages using the Latinate alphabet, Arabic is read from right to left and, most relevant for our purpose, is formed in cursive script in which clear spaces seldom exist between letters in words, even when printed formally. In addition, the physical shapes of Arabic letters also vary within the same typeface (see Al Jabry, 2015), depending on their position within a word, and these variations increase the total number of forms of Arabic letters to more than 100 (see also Jordan et al., 2010, 2014, 2015; Paterson et al., 2015). Thus, the physical appearance of Arabic differs substantially from stimuli formed in English, and other languages using the Latinate alphabet, and so Arabic offers a distinctive test of the generalizability of the print personality phenomenon. Accordingly, the purpose of this study is to provide the first investigation into whether fluent readers of Arabic attribute different personalities to different Arabic typefaces. If this attribution were found, it would provide a strong indication that the attribution of personalities to printed typefaces is a universal component of language perception, with important implications for understanding how humans generally process written language.

## Methods

### Ethics statement

This study was carried out in accordance with the recommendations of the Research Ethics Committee at Zayed University, with written informed consent from all participants, in accordance with the Declaration of Helsinki. The protocol was approved by the Research Ethics Committee at Zayed University.

### Participants

Participation in the experiment was invited by advertising around the campus at Zayed University and across the local area. Our primary criterion for inclusion was fluent reading of Arabic text by native adult Arabic readers but literacy varies enormously amongst the native population of the United Arab Emirates and the better readers of Arabic are often female. Accordingly, all applicants (male and female) were first screened for their reading ability (including vocabulary tests and reading rates; see Patching and Jordan, 2005a,b; Jordan et al., 2016) and those selected were chosen because they satisfied the requirements for fluent Arabic reading ability and satisfied additional criteria for visual ability (clearly, the experiment depended on visual perception). Following this procedure, 28 fluent, native-Arabic readers, with an age range of 18–28, participated in the experiment. All participants were female, and all had normal or corrected-to-normal vision, as determined by Bailey-Lovie (Bailey and Lovie, 1980) and Pelli-Robson (Pelli et al., 1988) assessments (see Jordan et al., 2011).

### Stimuli

Eleven different Arabic typefaces (see Table 1) used commonly in Arabic society were each presented as an Arabic sentence containing all letters of the alphabet (sentences such as this are known as *pangrams*). Each typeface was presented in 14-point. Participants were asked to rate the applicability of each of 20 different personality characteristics for each typeface (see Table 2), using a 1–7 scale (1 = *not at all*, 7 = *very*).

**Table 1:**
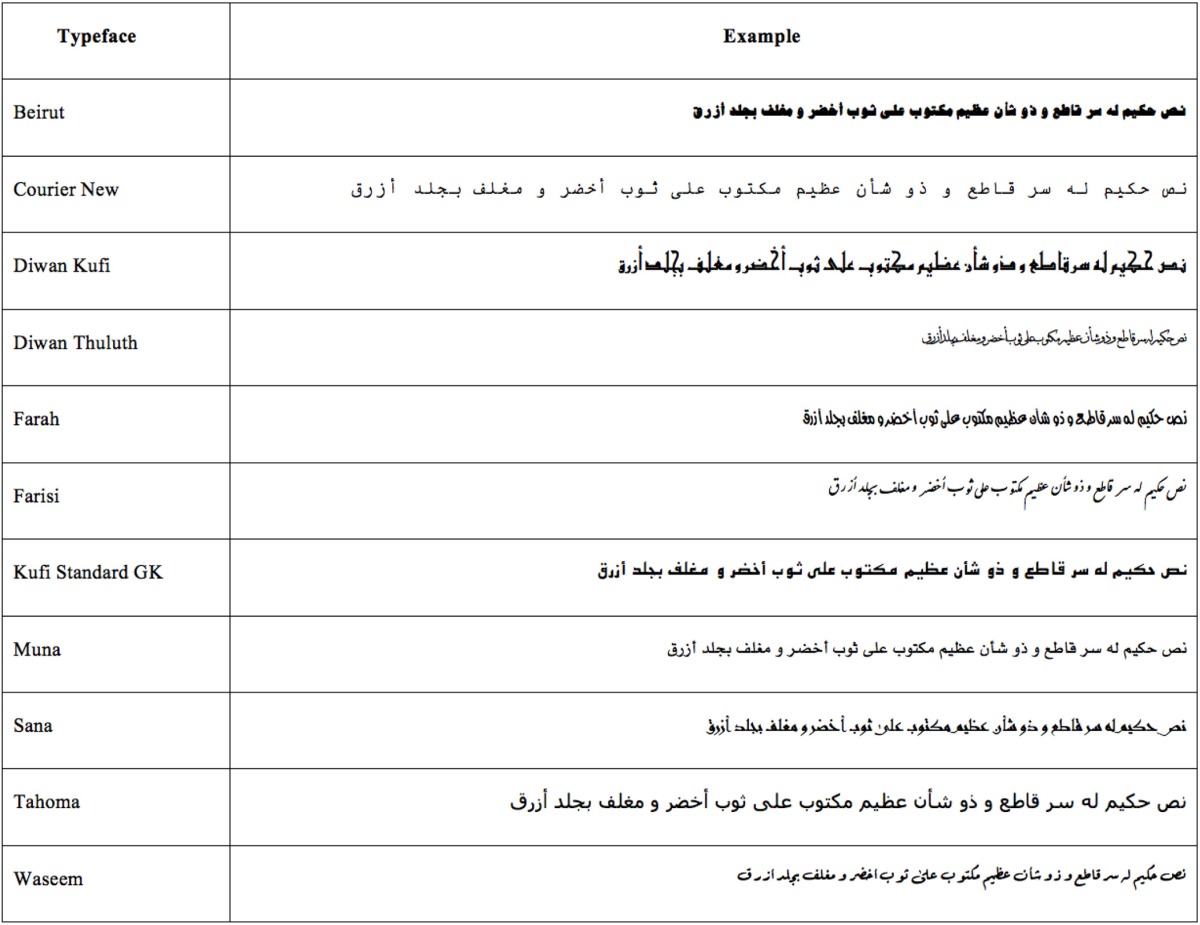
The typefaces used in this study.

**Table 2:** The print personality characteristics used in this study.

**Personality characteristic**
Cheap
Cold
Confident
Dignified
Elegant
Feminine
Formal
Friendly
Inviting
Loud
Masculine
Playful
Pretentious
Professional
Relaxed
Scholarly
Serious
Sloppy
Straight
Warm

### Design and procedure

Typeface was a within-participant independent variable and rating of the personality characteristics was the dependent variable. Each participant was given a booklet containing 11 separate sheets of paper with each sheet containing the pangram in one of the 11 different typefaces. Each sheet also contained the list of 20 personality characteristics which were listed in reverse order for half of the participants to counteract effects of response bias and fatigue. The sheets in each booklet were arranged in a different random order. Instructions were given orally to participants who were asked to make their ratings carefully. Each participant took ~20 min to complete the task.

## Results

For each typeface, the mean rating (*M*) for each personality characteristic and the standard deviation of these ratings (*SD*) are shown in Table 3. For each personality characteristic, a repeated-measures one-way analysis of variance (ANOVA) with the factor of Typeface was conducted to determine whether each characteristic was rated differently across different typefaces. The results of these analyses are shown in Table 4. Subsequent pair-wise comparisons between typefaces for each personality characteristic were performed using Bonferroni-corrected *t*-tests.

**Table 3:** For each typeface, Mean Rating (*M*) and Standard Deviation (*SD*) are shown for each personality characteristic.

**Typeface**		**Cheap**	**Cold**	**Confident**	**Dignified**	**Elegant**	**Feminine**	**Formal**	**Friendly**	**Inviting**	**Loud**	**Masculine**	**Playful**	**Pretentious**	**Professional**	**Relaxed**	**Scholarly**	**Serious**	**Sloppy**	**Straightforward**	**Warm**
Beirut	*M*	2.9	3.8	4.0	3.9	3.0	2.5	3.9	3.2	2.7	3.6	4.0	2.8	3.1	3.4	2.8	4.3	4.2	3.3	4.6	3.3
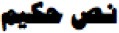	*SD*	2.3	2.1	1.9	1.9	1.8	1.6	2.1	1.5	1.5	2.5	2.1	1.7	1.6	1.9	1.5	2.0	1.7	2.1	2.1	1.7
Courier New	*M*	2.1	4.1	5.1	4.6	4.0	3.0	5.3	3.6	3.4	3.0	3.9	2.6	4.0	4.4	3.6	5.1	5.1	2.2	5.9	3.7
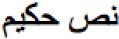	*SD*	1.7	2.3	1.5	1.8	2.1	1.7	1.9	1.7	1.9	2.2	2.0	1.8	1.8	1.9	1.9	1.8	1.7	1.5	1.5	2.0
Diwan Kufi	*M*	1.6	3.3	5.6	4.9	5.3	3.9	4.3	3.4	4.5	2.9	4.1	2.7	5.4	5.0	3.3	3.5	3.9	2.5	4.2	3.2
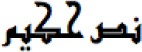	*SD*	1.3	2.2	1.8	2.0	1.8	2.4	2.1	2.1	2.0	1.9	2.3	2.1	2.1	2.0	2.1	2.3	2.0	1.8	1.8	2.1
Diwan Thuluth	*M*	2.1	3.6	5.4	5.2	5.2	4.2	3.9	3.5	4.8	3.4	3.4	2.8	5.1	4.5	3.3	3.0	3.7	2.7	3.6	3.8
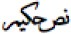	*SD*	1.7	2.0	1.9	1.6	1.9	1.8	2.2	2.0	1.8	2.0	2.0	1.9	2.0	2.1	2.0	1.9	2.0	2.0	2.3	2.3
Farah	*M*	2.6	3.1	4.0	3.6	3.7	3.6	2.7	4.5	3.4	2.9	2.8	3.6	3.7	3.4	4.2	3.3	2.5	2.7	4.6	3.6
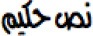	*SD*	2.0	1.9	1.7	1.9	1.9	1.9	1.5	1.6	2.0	2.1	1.7	2.0	2.0	1.9	1.8	2.0	1.4	1.7	2.0	1.6
Farisi	*M*	2.4	3.3	5.4	4.9	5.2	4.1	3.6	3.8	4.5	2.8	3.8	3.2	5.1	4.5	4.6	3.4	3.6	2.4	3.4	4.0
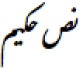	*SD*	1.9	2.2	1.8	1.9	1.8	2.2	2.1	1.7	2.0	1.7	1.9	1.5	1.7	2.0	2.1	2.1	2.3	1.7	1.6	1.9
Kufi Standard GK	*M*	2.2	2.7	4.7	5.0	4.5	2.9	4.7	3.8	4.1	2.4	4.4	2.1	4.2	4.6	4.0	4.8	5.2	2.1	6.3	3.7
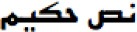	*SD*	1.6	1.5	1.5	1.6	2.0	1.6	1.8	1.8	1.8	1.6	2.0	1.0	1.7	1.6	1.8	2.0	1.3	1.2	1.1	1.8
Muna	*M*	1.5	2.7	6.0	6.3	5.7	3.9	6.6	5.3	5.2	1.5	4.2	2.0	4.0	5.5	4.1	6.3	6.1	1.7	6.7	5.1
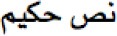	*SD*	1.3	1.8	1.4	1.0	1.7	2.0	0.7	1.5	1.5	0.9	2.0	1.2	1.9	1.9	2.2	1.0	1.4	1.3	0.7	1.9
Sana	*M*	1.6	3.2	5.1	5.0	5.1	3.4	4.6	4.1	4.9	2.4	4.0	2.3	5.0	5.0	3.4	3.7	4.0	2.4	4.1	3.7
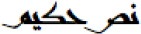	*SD*	1.1	1.5	1.7	1.7	2.0	2.3	2.3	2.0	2.0	1.4	2.2	1.5	2.0	1.9	2.1	2.2	2.1	1.8	1.7	1.9
Tahoma	*M*	2.9	3.8	4.3	4.9	3.5	3.0	4.0	4.4	3.2	2.8	3.1	2.7	3.1	3.7	4.6	4.8	3.9	3.0	6.2	4.0
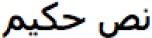	*SD*	2.0	2.2	2.1	1.4	2.1	2.0	2.2	2.0	2.2	2.1	1.9	1.8	2.2	2.2	1.9	1.9	2.3	1.9	1.3	2.3
Waseem	*M*	1.6	2.5	5.3	5.1	5.4	4.3	3.8	5.3	5.2	2.1	3.3	2.7	4.7	4.9	5.0	3.5	4.0	2.5	4.2	4.7
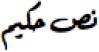	*SD*	1.1	1.6	1.7	1.5	1.5	2.1	2.0	1.5	1.6	1.6	2.1	1.8	1.8	2.1	1.8	1.9	2.2	1.5	2.1	1.9

**Table 4:** Results of analyses of variance comparing the ratings of each personality characteristic across the typefaces.

**Personality characteristic**	***df*_Typeface_**	***df*_Error_**	***F***	***p*<**
Cheap	5.85	158.00	3.39	0.005
Cold	6.47	174.61	2.93	0.010
Confident	10.00	270.00	4.61	0.001
Dignified	6.19	167.20	5.91	0.001
Elegant	10.00	270.00	7.80	0.001
Feminine	6.19	167.15	3.52	0.005
Formal	5.86	158.32	8.42	0.001
Friendly	10.00	270.00	4.76	0.001
Inviting	10.00	270.00	6.68	0.001
Loud	6.12	165.25	3.62	0.005
Masculine	10.00	270.00	2.69	0.005
Playful	5.96	161.00	2.68	0.020
Pretentious	6.56	177.20	5.97	0.001
Professional	6.01	162.25	3.71	0.005
Relaxed	10.00	260.00	3.86	0.001
Scholarly	5.78	156.11	9.03	0.001
Serious	5.27	142.24	8.00	0.001
Sloppy	10.00	270.00	2.14	0.050
Straightforward	5.27	142.39	17.28	0.001
Warm	10.00	270.00	2.61	0.010

*Degrees of freedom are Greenhouse-Geisser corrected where appropriate*.

**Figure 1:**
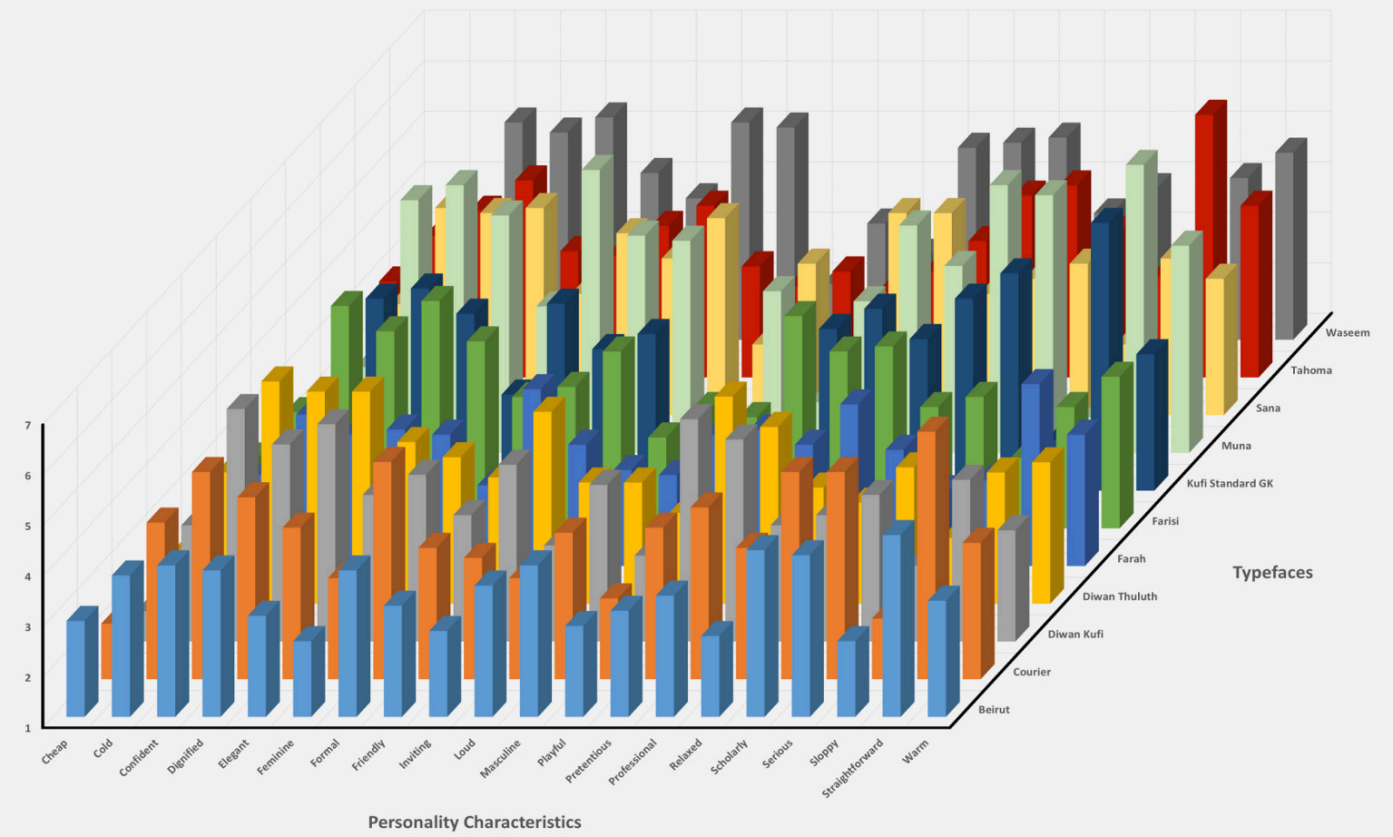
The rating of personality characteristics for each typeface.

As indicated in Table 4 (see also Figure 1), typefaces produced widespread effects on the ratings of each of the 20 personality characteristics and these effects were examined more closely using pair-wise Bonferroni-corrected *t*-tests to determine precisely, for each personality characteristic, which typefaces produced significantly different ratings. These tests revealed multiple significant differences between typefaces for each personality characteristic [the full list of these *post-hoc* pairwise comparisons is provided in Appendix 1 (Supplementary Material)]. For example, Muna was rated as more confident (*p* < 0.005) and more dignified (*p* < 0.001) than Farah and more elegant than Tahoma (*p* < 0.005). In a similar way, Courier New was rated as more formal (*p* < 0.001) and more serious (*p* < 0.001) than Farah and more straightforward than Farisi (*p* < 0.001).

## Discussion

The purpose of this study was to determine whether fluent readers of Arabic attribute different personalities to different Arabic typefaces. Previous research on this issue has used only English stimuli and so the generality of the phenomenon to other languages, especially those that are visually very different from English, was unknown. The results of the study are clear and show, for the first time, that Arabic typefaces can indeed convey meaning (print personalities) generated by a typeface's visual structure, such that each of the typefaces used in this study produced a different pattern of personality characteristics. Thus, despite the substantial differences that exist between the visual forms of written Arabic and English, it is now apparent that the surface details of both languages are capable of producing print personalities, and these results provide important indications of the generalizability of the phenomenon and the nature of the formation of personality attributions. In particular, when considering previous findings obtained using typefaces in English, it was unclear whether the formation of print personalities relied on languages in which letters are separated by clear spacing and are physically consistent when appearing in different locations in words. But unlike languages such as English that use the Latinate alphabet, Arabic is formed in cursive script in which clear spaces seldom exist between letters in words, and the physical shapes of Arabic letters vary considerably within the same typeface, depending on their position within the word. Consequently, as Arabic is also capable of generating semantic information from typefaces, it seems that the surface details of print that contribute to the formation of print personalities are not restricted to the well-structured letter-by-letter format of English.

It is too early to determine what precise features of a written language are used to generate print personalities but it is not unreasonable to speculate which aspects are likely to underlie this generality across Arabic and English. In particular, while these two languages differ in the discernibility of their individual letters, both languages contain letter strokes which have consistent width, weight, seriation, and curvature within a typeface, and these aspects of print can be perceived irrespective of whether the spacing and visual form of letters in languages are consistent (as in Arabic vs. English). Moreover, these visual elements may be encoded as supraletter features in which the surface details of letter strokes extend as a unit across two or more letters, thus producing print personalities in languages as visually diverse as English and Arabic. Indeed, for Arabic, supraletter features may be easier to process than separate letter-based codes due to the lack of spacing and physical consistency between letters in this language (see Jordan et al., 2010, 2014, 2015; Paterson et al., 2015). Accordingly, information from letter strokes, rather than individual letters, may be important for the attribution of print personalities to typefaces.

But how might the surface detail of typefaces generate particular personality characteristics? One way is that readers encounter typefaces regularly in specific circumstances (e.g., books, newspapers, formal certificates, places of worship, etc.) and these associations help define the personality characteristics attributed to a typeface. For example, in Arabic, serious text books and newspapers are often printed using Muna, and this typeface was rated highly in our study for straightforward, formal, and scholarly. In a similar vein, Sana and Diwan Kufi are often seen in Mosque designs, in poetry, and in formal certificates, and both these typefaces were rated highly for confident, elegant, pretentious, and dignified. So the context in which typefaces are regularly encountered in Arabic may exert at least some influence on the personalities attributed to print, perhaps through processes of perceptual categorization which rely heavily on the associative property of memory (Oppenheimer and Frank, 2008; see also Laliberte, 1987).

A second, and perhaps more enduring, source of influence is the generation of print personalities in a manner that is similar to the way in which humans personify objects generally in everyday life. Indeed, research on the personification of objects, where objects that are not directly related to a personal characteristic nonetheless evoke that characteristic, suggests that the tendency for people to ascribe characteristics to objects is quite commonplace (e.g., Boroditsky et al., 2003). Gal and Wilkie (2010), for example, investigated the way in which people ascribe gender to items of furniture, and found that observers were more likely to assign male characteristics to furniture with sharp edges, such as a square table, and female characteristics to furniture with round edges, such as a round table. Comparable perceptions were shown by participants in our experiment who consistently rated some Arabic typefaces as either more masculine or more feminine. For example, Kufi standard (see Table 3) had the highest rating for masculine but also the lowest rating for feminine. Waseem, on the other hand, had the highest rating for feminine but also had one of the lowest ratings for masculine. Thus, the consistency with which participants rated the masculine and feminine qualities of typefaces in our study may be an indication of the pervasiveness of gender as a framework for how we conceptualize elements in our visual world. But less explicable are the links between directly perceivable features of typefaces and other more abstract connotative dimensions such as scholarly, confident, friendly, and playful. The ease and consistency with which participants were able to do so suggests that perceptual categorization of the personalities of typefaces can be derived even for concepts that are rather abstractly linked to the physical qualities of print.

The influence of semantic information derived from the surface details of print is still largely ignored in the literature on word recognition and reading. Indeed, theories of word recognition and reading generally address only those aspects of printed words that are used to access the appropriate lexical representations (e.g., Rayner, 2009; Davis, 2010), and the surface details of words are of little relevance in this matter or are discarded at an early stage in visual processing. This is not to say that the visual appearances of typefaces do not affect lexical access (e.g., they can make stimuli harder or easier to read, due to such things as crowding; Levi, 2008) but, rather, that typefaces provide no extra source of information for the reading experience. However, this approach excludes the previous evidence (obtained in English) that typefaces can provide an additional source of semantic information and, from the indications provided by the present study, can be obtained across languages with very different visual characteristics. Indeed, some research suggests that the semantic information provided by typefaces actually affects access to the linguistic meaning of words (at least in English). Lewis and Walker (1989), for example, found that inconsistency between a typeface's visual appearance (e.g., light or heavy) and the actual meaning of a word (e.g., a light or heavy animal) produced longer reaction times in an animal categorization task relative to when typeface and word meaning were consistent. In a similar vein, Foltz et al. (1984) found that interference and facilitation effects were apparent when the size of a typeface was either congruent or incongruent with the size represented linguistically by the word (e.g., elephant vs. mouse; see also Seymour and Jack, 1978; Walker et al., 1986; Song and Schwarz, 2008). Thus, typographic features of words appear to access a semantic code sufficiently rapidly to interact at some point with the derivation a word's linguistic meaning. It seems highly likely, from the present findings using Arabic, that this influence of the visual appearance of typefaces on the semantic information provided by words may extend across a range of languages, indicating that this influence is not tied to the visual appearance of just one alphabet or, indeed, one language.

Finally, it is worth underscoring the fact that the participants who took part in this study were selected for their fluent Arabic reading ability, and it remains to be seen whether similar findings are obtained with other reading groups. For example, it may be the case that perception of print personalities is closely associated with reading ability (and experience generally with a written language), and so readers with lower reading ability (and less experience) may show different effects from those we observed. Moreover, the criterion for reading ability in the present study produced a participant sample which was all female. Although some evidence suggests that gender differences can occur in the basic abilities of beginning (child) readers of Arabic (e.g., Mohamed et al., 2010; Emam et al., 2014), there is currently no evidence to suggest that adult females perform differently from adult males in the task of assigning personalities to Arabic typefaces. Indeed, while it may be tempting to think that a random selection of each gender for an experiment provides clear information on the influence of gender on performance, many other individual differences and similarities will naturally exist within the participant sample. Accordingly, properly-conducted, reading ability and cross-gender comparisons are needed to address these issues, and these may well follow in future research on perception of print personalities in Arabic, and other languages.

## Author contributions

TJ and MS conceived the experiment. TJ, MS, and MA designed the experiment. AA, HY, MA, and NA ran the experiment. HY, MA, and EH analyzed the data. TJ, EH, and MS wrote the paper.

### Conflict of interest statement

The authors declare that the research was conducted in the absence of any commercial or financial relationships that could be construed as a potential conflict of interest.

## References

[B1] Al Jabry S. A. (2015). From Script to Font, Arabic's Struggle to Reclaim its Calligraphic Beauty. Dubai: The National.

[B2] Bailey I. L. Lovie J. E. (1980). The design and use of a new near-vision chart. Am. J. Optom. Physiol. Opt. 57, 378–387. 10.1097/00006324-198006000-00011 7406006

[B3] Bartram D. (1982). The perception of semantic quality in type: differences between designers and non-designers. Inf. Des. J. 3, 30–37. 10.1075/idj.3.1.04bar

[B4] Boroditsky L. Schmidt L. Phillips W. (2003). Sex, syntax, and semantics, in Language in Mind: Advances in the Study of Language and Cognition, eds Gentner D. Goldin-Meadow S. (Cambridge, MA: MIT Press), 61–80.

[B5] Brumberger E. (2003). The rhetoric of typography: the persona of typeface and text. Tech. Commun. 50, 206–223.

[B6] Davis C. J. (2010). The spatial coding model of visual word identification. Psychol. Rev. 117, 713–758. 10.1037/a0019738 20658851

[B7] Doyle J. Bottomley P. (2004). Font appropriateness and brand choice. J. Bus. Res. 57, 873–880. 10.1016/S0148-2963(02)00487-3

[B8] Emam M. Kazem A. Al-Said T. Al-Maamary W. Al-Mandhari R. (2014). Variations in Arabic reading skills between normally achieving and at risk for reading disability students in second and fourth grades. Rev. Eur. Stud. 6:17 10.5539/res.v6n3p17

[B9] Fazio R. H. (2001). On the automatic activation of associated evaluations: an overview. Cogn. Emotion 13, 115–141. 10.1080/02699930125908

[B10] Foltz G. S. Poltrock S. C. Potts G. R. (1984). Mental comparison of size and magnitude: size congruity effects. J. Exp. Psychol. Learn. Mem. Cogn. 10, 442–453. 10.1037/0278-7393.10.3.442 6235311

[B11] Gal D. Wilkie J. (2010). Real men don't eat quiche: regulation of gender-expressive choices by men. Soc. Psychol. Pers. Sci. 1, 291–301. 10.1177/1948550610365003

[B12] Jordan T. R. Almabruk A. A. A. Gadalla E. A. McGowan V. A. White S. J. Abedipour L.. (2014). Reading direction and the central perceptual span: evidence from Arabic and English. Psychon. Bull. Rev. 21, 505–511. 10.3758/s13423-013-0510-4 24065283

[B13] Jordan T. R. Dixon J. McGowan V. A. Kurtev S. Paterson K. B. (2016). Fast and slow readers and the effectiveness of the spatial frequency content of text: evidence from reading times and eye movements. J. Exp. Psychol. Human Percept. Perform. 42, 1066–1071. 10.1037/xhp0000234 27123680

[B14] Jordan T. R. McGowan V. A. Paterson K. B. (2011). Out of sight, out of mind: the rarity of assessing and reporting participants' visual abilities when studying perception of linguistic stimuli. Perception 40, 873–876. 10.1068/p6940 22128559

[B15] Jordan T. R. Paterson K. B. Almabruk A. A. A. (2010). Revealing the superior perceptibility of words in Arabic. Perception 39, 426–428. 10.1068/p6637 20465177

[B16] Jordan T. R. Redwood M. Patching G. R. (2003). Effects of form familiarity on perception of words, pseudowords and nonwords in the two cerebral hemispheres. J. Cogn. Neurosci. 15, 537–548. 10.1162/089892903321662921 12803965

[B17] Jordan T. R. Sheen M. Al Jassmi M. Paterson K. B. (2015). A new demonstration of the illusory letters phenomenon: graphemic restoration in Arabic word perception. Perception 44, 215–218. 10.1068/p7885 26561973

[B18] Juhasz B. J. Liversedge S. P. White S. J. Rayner K. (2006). Binocular coordination of the eyes during reading: word frequency and case alternation affect fixation duration but not binocular disparity. Q. J. Exp. Psychol. 59, 1614–1625. 10.1080/17470210500497722 16873112

[B19] Juni S. Gross J. S. (2008). Emotional and persuasive perception of fonts. Percept. Mot. Skills 106, 35–42. 10.2466/pms.106.1.35-42 18459353

[B20] Kostelnick C. (1990). The rhetoric of text design in professional communication. Tech. Writing Teach 17, 189–202.

[B21] Laliberte J. (1987). La typographie moderne: consequence de la revolution industrielle? Commun. Lang. 72, 60–76. 10.3406/colan.1987.974

[B22] Levi D. M. (2008). Crowding-an essential bottleneck for object recognition: a mini-review. Vis. Res. 48, 635–654. 10.1016/j.visres.2007.12.009 18226828 PMC2268888

[B23] Lewis C. Walker P. (1989). Typographic influences on reading. Br. J. Psychol. 80, 241–257. 10.1111/j.2044-8295.1989.tb02317.x 2736343

[B24] Mackiewicz J. (2004). What technical writing students should know about typeface personality. J. Tech. Writing Commun. 34, 113–131. 10.2190/NMDQ-XBVH-Q79J-M749

[B25] Mackiewicz J. Moeller R. (2004). Why people perceive typefaces to have different personalities, in Paper Presented at the International Professional Communication Conference (Minneapolis, MN).

[B26] Mayall K. A. Humphreys G. W. Olson A. (1997). Disruption to word or letter processing? The origins of case-mixing effects. J. Exp. Psychol. Learn. Mem. Cogn. 23, 1275–1286. 10.1037/0278-7393.23.5.1275 9293635

[B27] McCarthy M. Mothersbaugh D. (2002). Effects of typographic factors in advertising-based persuasion: a general model and initial empirical tests. Psychol. Mark. 19, 663–691. 10.1002/mar.10030

[B28] Mohamed W. Elbert T. Landerl K. (2010). The development of reading and spelling abilities in the first 3 (three) years of learning Arabic. Reading Writing 24, 1043–1060. 10.1007/s11145-010-9249-8

[B29] Moret-Tatay C. Perea M. (2011). Do serifs provide an advantage in the recognition of written words? J. Cogn. Psychol. 23, 619–624. 10.1080/20445911.2011.546781

[B30] Oppenheimer D. M. Frank M. C. (2008). A rose in any other font would not smell as sweet: effects of perceptual fluency on categorization. Cognition 106, 1178–1194. 10.1016/j.cognition.2007.05.010 17618616

[B31] Parker R. C. (1997). Looking Good in Print. Research Triangle Park, NC: Ventana Communications Group, Inc.

[B32] Patching G. R. Jordan T. R. (2005a). Assessing the role of different spatial frequencies in word perception by good and poor readers. Mem. Cognit. 33, 961–971. 10.3758/BF03193205 16496718

[B33] Patching G. R. Jordan T. R. (2005b). Spatial frequency sensitivity differences between adults of good and poor reading ability. Invest. Ophthalmol. Vis. Sci. 46, 2219–2224. 10.1167/iovs.03-1247 15914644

[B34] Paterson K. B. Almabruk A. A. A. McGowan V. A. White S. J. Jordan T. R. (2015). Effects of word length on eye movement control: the evidence from Arabic. Psychon. Bull. Rev. 22, 1443–1450. 10.3758/s13423-015-0809-4 25690581

[B35] Pelli D. G. Robson J. G. Wilkins A. J. (1988). The design of a new letter chart for measuring contrast sensitivity. Clin. Vis. Sci. 2, 187–199.

[B36] Perea M. Vergara-Martínez M. Gomez P. (2015). Resolving the locus of cAsE aLtErNaTiOn effects in visual word recognition: evidence from masked priming. Cognition 142, 39–43. 10.1016/j.cognition.2015.05.007 26010560

[B37] Poffenberger A. T. Franken R. B. (1923). A study of the appropriateness of typefaces. J. Appl. Psychol. 7, 312–329. 10.1037/h0071591

[B38] Rayner K. (2009). Eye movements and attention in reading, scene perception, and visual search. Q. J. Exp. Psychol. 62, 1457–1506. 10.1080/17470210902816461 19449261

[B39] Sassoon R. (1993). Through the eyes of a child: perception and type design, in Computers and Typography, ed Sassoon R. (Oxford: Intellect Books), 150–177.

[B40] Secrest J. M. (1947). Personalities in type designs. Printers Ink 218, 52–53.

[B41] Seymour P. H. Jack M. V. (1978). Effects of visual familiarity on “same” and “different” decision processes. Q. J. Exp. Psychol. 30, 455–469. 10.1080/00335557843000052 693789

[B42] Song H. Schwarz N. (2008). If it's hard to read, it's hard to do: processing fluency affects effort prediction and motivation. Psychol. Sci. 19, 986–988. 10.1111/j.1467-9280.2008.02189.x 19000208

[B43] Spiekermann E. Ginger E. M. (1993). Stop Stealing Sheep and Find Out How Type Works. Mountain View, CA: Adobe.

[B44] Striver I. (2001). Type Rules! Cincinnati, OH: North Light Books.

[B45] Sushan R. Wright D. (1989). Desktop Publishing by Design. Redmond, WA: Microsoft Press.

[B46] Tantillo J. Di Lorenzo-Aiss J. Mathisen R. E. (1995). Quantifying perceived differences in type styles: an exploratory study. Psychol. Mark. 12, 447–457. 10.1002/mar.4220120508

[B47] Walker P. Smith S. Livingston A. (1986). Predicting the appropriateness of a typeface on the basis of its multi-modal features. Inform. Des. J. 5, 29–42. 10.1075/idj.5.1.02wal

[B48] Zachrisson B. (1965). Studies in the Legibility of Printed Text. Stockholm: Almqvist & Wiksell.

