# Supplementary material for: What's in a Typeface? Evidence of the Existence of Print Personalities in Arabic
Source: Front Psychol. 2017 Oct 18;8:1229. doi: 10.3389/fpsyg.2017.01229 (PMC5651518; doi:10.3389/fpsyg.2017.01229)
Supplement: Supplementary file 1 [file DataSheet1.pdf]

Appendix 1

For each personality characteristic, significant pair-wise comparisons between typefaces are reported, together with their mean difference and Standard Error (SE).

Personality Characteristic	Pairwise Contrast	Mean Difference	SE	*p*<
Cheap	Tahoma > Muna	1.39	0.34	.020
Cold	Courier New > Kufi Standard	1.46	0.34	.020
Confident	Muna > Beirut	1.96	0.48	.050
	Muna > Tahoma	1.64	0.43	.050
	Muna > Farah	1.93	0.41	.005
	Diwan Kufi > Farah	1.54	0.38	.050
Dignified	Muna > Sana	1.25	0.33	.050
	Sana > Farah	1.43	0.38	.050
	Muna > Beirut	2.32	0.38	.001
	Muna > Courier New	1.64	0.34	.005
	Muna > Tahoma	1.32	0.21	.001
	Muna > Farah	2.68	0.41	.001
	Muna > Kufi Standard	1.21	0.32	.050
	Waseem > Farah	1.57	0.39	.050
	Kufi Standard > Beirut	1.11	0.29	.050
Elegant	Farisi > Beirut	2.18	0.36	.001
	Sana > Beirut	2.11	0.49	.020
	Muna > Beirut	2.68	0.49	.001
	Diwan Thuluth > Beirut	2.15	0.36	.001

	Diwan Kufi > Beirut	2.32	0.43	.002
	Waseem > Beirut	2.36	0.38	.001
	Muna > Tahoma	2.18	0.45	.005
	Muna > Farah	2.00	0.37	.002
	Diwan Kufi > Tahoma	1.82	0.42	.020
	Diwan Kufi > Farah	1.64	0.42	.050
	Waseem > Farah	1.68	0.41	.020
	Farisi > Beirut	1.64	0.38	.020
Feminine	Diwan Thuluth > Beirut	1.71	0.37	.010
	Waseem > Beirut	1.79	0.38	.005
	Muna > Beirut	2.75	0.42	.001
	Muna > Farisi	3.04	0.45	.001
	Muna > Sana	2.04	0.45	.010
	Sana > Farah	1.91	0.46	.020
	Muna > Tahoma	2.57	0.45	.001
	Muna > Farah	3.94	0.35	.001
Formal	Muna > Diwan Thuluth	2.72	0.46	.001
	Muna > Diwan Kufi	2.36	0.42	.001
	Muna > Waseem	2.79	0.41	.001
	Muna > Kufi Standard	1.94	0.34	.001
	Courier New > Farah	2.58	0.46	.001
	Kufi Standard > Farah	2.00	0.47	.020
	Muna > Beirut	2.04	0.44	.005
Friendly	Waseem > Beirut	2.04	0.36	.001
	Waseem > Farisi	1.50	0.37	.050

	Muna > Courier New	1.62	0.39	.020
	Muna > Diwan Thuluth	1.79	0.46	.050
	Muna > Diwan Kufi	1.82	0.43	.020
	Waseem > Courier New	1.62	0.39	.020
	Waseem > Diwan Thuluth	1.79	0.46	.050
	Waseem > Diwan Kufi	1.82	0.46	.050
	Farisi > Beirut	1.82	0.40	.010
	Sana > Beirut	2.18	0.48	.010
	Muna > Beirut	2.50	0.37	.001
	Diwan Thuluth > Beirut	2.07	0.37	.001
	Diwan Kufi > Beirut	1.75	0.47	.050
Inviting	Waseem > Beirut	2.46	0.37	.001
	Muna > Courier New	1.82	0.45	.050
	Muna > Tahoma	2.04	0.45	.010
	Muna > Farah	1.79	0.48	.050
	Waseem > Courier New	1.79	0.43	.020
	Waseem > Tahoma	2.00	0.49	.050
	Beirut > Muna	2.18	0.49	.010
Loud	Farisi > Muna	1.36	0.35	.050
	Diwan Thuluth > Muna	1.89	0.43	.010
	Diwan Kufi > Muna	1.43	0.36	.050
Masculine	Kufi Standard > Farah	1.57	0.41	.050
Playful	Farisi > Muna	1.21	0.31	.050
	Farah > Muna	1.61	0.41	.050

	Farah > Kufi Standard	1.46	0.38	.050
	Farisi > Beirut	2.01	0.32	.001
	Sana > Beirut	1.86	0.42	.010
	Diwan Thuluth > Beirut	1.96	0.38	.002
Pretentious	Diwan Kufi > Beirut	2.29	0.51	.010
	Waseem > Beirut	1.60	0.34	.005
	Farisi > Tahoma	2.04	0.52	.050
	Diwan Kufi > Tahoma	2.32	0.55	.020
Professional	Muna > Beirut	2.07	0.34	.001
	Sana > Farah	1.64	0.43	.050
	Waseem > Beirut	2.33	0.38	.001
Relaxed	Waseem > Diwan Thuluth	1.78	0.42	.020
	Waseem > Diwan Kufi	1.78	0.41	.020
	Muna > Beirut	2.00	0.47	.020
	Muna > Farisi	2.89	0.49	.001
	Muna > Sana	2.54	0.47	.002
	Muna > Tahoma	1.46	0.31	.005
	Muna > Farah	3.00	0.45	.001
Scholarly	Muna > Diwan Thuluth	3.21	0.43	.001
	Muna > Diwan Kufi	2.79	0.48	.001
	Muna > Waseem	2.79	0.41	.001
	Courier New > Diwan Thuluth	2.11	0.47	.010
	Tahoma > Diwan Thuluth	1.75	0.45	.050

	Muna > Beirut	1.89	0.46	.050
	Muna > Farisi	2.46	0.53	.010
	Muna > Sana	2.07	0.51	.050
	Sana > Farah	1.52	0.40	.050
	Muna > Tahoma	2.14	0.45	.005
Serious	Muna > Farah	3.59	0.37	.001
	Muna > Diwan Thuluth	2.36	0.43	.001
	Muna > Diwan Kufi	2.21	0.46	.005
	Muna > Waseem	2.11	0.45	.005
	Courier New > Farah	2.59	0.37	.001
	Kufi Standard > Farah	2.74	0.38	.001
Sloppy	Tahoma > Muna	1.25	0.31	.050
	Muna > Beirut	2.07	0.41	.002
	Tahoma > Beirut	1.57	0.36	.010
	Kufi Standard > Beirut	1.64	0.34	.005
	Muna > Farisi	3.32	0.35	.001
	Courier New > Farisi	2.54	0.39	.001
	Tahoma > Farisi	2.82	0.39	.001
	Kufi Standard > Farisi	2.89	0.38	.001
Straightforward	Muna > Sana	2.61	0.37	.001
	Courier New> Sana	1.82	0.43	.020
	Tahoma> Sana	2.11	0.40	.002
	Kufi Standard > Sana	2.18	0.39	.001
	Muna > Farah	2.11	0.40	.002
	Muna > Diwan Thuluth	3.14	0.45	.001
	Muna > Diwan Kufi	2.54	0.38	.001

	Muna > Waseem	2.50	0.44	.001
	Courier New > Diwan Thuluth	2.36	0.54	.010
	Tahoma > Farah	1.61	0.37	.010
	Tahoma > Diwan Thuluth	2.64	0.47	.001
	Tahoma > Diwan Kufi	2.04	0.42	.005
	Tahoma > Waseem	2.00	0.44	.010
	Kufi Standard > Farah	1.68	0.33	.005
	Kufi Standard > Diwan Thuluth	2.71	0.43	.001
	Kufi Standard > Diwan Kufi	2.11	0.47	.010
	Kufi Standard > Waseem	2.07	0.43	.005
Warm	Muna > Diwan Kufi	1.89	0.46	.020

Note: Pair-wise comparisons were performed using Bonferroni-corrected *t*-tests.
